# Do negative emotions in social advertising really work? Confrontation of classic vs. EEG reaction toward advertising that promotes safe driving

**DOI:** 10.1371/journal.pone.0233036

**Published:** 2020-05-15

**Authors:** Anna Borawska, Tomasz Oleksy, Dominika Maison

**Affiliations:** 1 Faculty of Finance, Economics and Management, University of Szczecin, Szczecin, Poland; 2 Faculty of Psychology, University of Warsaw, Warsaw, Poland; Tongii University, CHINA

## Abstract

Social campaigns are persuasive messages that attempt to communicate positive ideas and practices. One of the main challenges in designing effective social campaigns is the need to compete with other advertisements for viewers’ attention. One of the most widely used methods of drawing attention to social advertising is the use of negative emotions. However, the effectiveness of negative emotional appeals in social campaigns is still a topic of debates. The aim of the study was to use both declarative and neural (EEG) measures to examine whether increasing the intensity of negative emotions in a social campaign enhances its effectiveness linearly or only to a certain level (curvilinear relation). The experimental study was conducted (N = 62) with road safety campaign, using three different levels of negative emotional intensity. The results showed that even though advertising with the strongest negative stimuli evoked the strongest negative emotions, it had no significantly stronger influence on behavioral intention (driving less risky) than moderately negative stimuli. Moreover, neural reaction to the negative stimuli in advertising depended on driving style–people with risky driving style payed less attention to more threatening message (higher beta oscillations).

## Introduction

A social campaign is a persuasive message that aims to change attitudes or behavior in order to improve the welfare of individuals and society [1, 2]. Social campaigns attempt to communicate positive ideas and practices via mass media, social media and interpersonal communication (e.g. [3]). They concern various issues related to human life, such as the perception of national or sexual minorities (attitude change), eating habits, healthy lifestyles, safe driving or financial support for various charities (behavioral change).

Designing effective social campaigns poses a major challenge due to the complexity of the issues that they address. For example, receivers may hold both positive and negative attitudes toward a given topic; the more ambivalent the attitudes are, the lower the behavioral change intentions could be, as in the case of pro-environmental behavior or buying “green” products [4, 5]. Understanding the sources of this ambivalence may also be hampered by the discrepancy between explicit and implicit attitudes toward the issues presented, resulting from, among others, the need for social approval and unconscious cognitive or motivational processes [6].

Another challenge in creating a successful media campaign is the need to compete with other advertisements for viewers’ attention. Advertising clutter can make it difficult for a message to break through the daily information overload, especially as the topics covered by social campaigns are often more cognitively and emotionally demanding than typical commercial advertising communication (e.g. [7, 8]). Therefore, in order to create effective social campaign communication that promotes a desired social change, ambivalent attitudes must be overcome, and an adequate amount of recipients’ attention attracted.

One method of drawing attention to advertising is to use emotions. Driven by the need for visibility, social campaigns increasingly use emotions, especially negative ones, and even shocking messages [9–11]. Apart from drawing the audience’s attention, negative emotions are used in social advertising as a persuasion tool. Use of emotions is a way to change attitudes and behavior [12]. However, negative emotions are used in advertising with varying degrees of success, especially because they risk drawing attention away from the advertising message [13] and evoking viewers’ defense mechanisms [14].

Research in the field shows a lot of inconsistency, as some studies prove that the greater the intensity of the negative emotions, the greater the effectiveness of the social communication (e.g. [15–17]). Others show entirely different results (e.g. [18, 19]). As a consequence of the discrepancies in results regarding the role of negative emotions in social advertising, campaigns have been realized using two competing approaches for many years. The first assumes a linear correlation between the increase in negative emotions and advertising effectiveness [20, 21]. The second proposes a curvilinear relation [22] based on the assumption that increase in negative emotions can influence a positive change of attitudes and behaviors to a certain level. When this level is too high, the influence on attitudes and behaviors can decrease because of defense mechanism activation [23].

Most studies on negative emotions are based on declarations. In these studies, the effectiveness of ads is measured by questionnaires completed by respondents. However, it is possible that reactions toward negative emotions in advertising are partially unconscious (e.g. because of defense mechanisms, e.g. [24]), so declarative measures alone are not sufficient to investigate this problem. The solution to these problems may be to use brain activity measurements, such as fMRI [25, 26] or EEG [27, 28], which can capture more automatic and unconscious reactions to social campaigns as well as cognitive processes evoked by the ad. Therefore, the aim of the current study is to use both declarative and neural examinations to determine which of the following hypotheses is true: 1) increasing the intensity of negative emotions in advertising enhances its effectiveness linearly, or 2) increasing the intensity of negative emotions in advertising increases its effectiveness only to a certain level.

This research was conducted in the context of a road safety campaign, using three different levels of negative emotional intensity, from low to very violent depictions of accident victims. Given the fact that previous research has shown that being in a risk group can significantly alter reactions toward a social campaign (see [23], in the context of smokers vs. non-smokers), we also measured driving style as a factor potentially influencing the relationship between negative emotional appeals and the effectiveness of the campaign. We believe that by combining 1) the possibility to test a linear vs. curvilinear hypothesis using three levels of emotional messages with 2) declarative and neural measures, and (3) by examining the potential moderating role of individual behavioral styles, our study will contribute significantly to the research on the effectiveness of negative emotions in promoting behavioral change.

## The role of negative emotional appeals in persuasive advertisement campaigns

The pioneering research on negative appeals in persuasive communication was published in the 1950s [29] and it examined the effect of fear appeal on adopting dental hygiene recommendations. Since then, researchers have shown a lot of interest in how individuals perceive, process, and react to messages that contain threatening information. The use of such emotions in social campaigns is widely described in the literature, and the main reasons for their popularity are their ability to draw an audience’s attention and increase engagement in order to mobilize people to take specific actions [30]. Moreover, negative messages are also perceived as more reliable and informative [31].

However, there is still an ongoing discussion as to the relationship between the intensity of negative emotions and advertising effectiveness, i.e. to what level of intensity of negative emotions (regardless of their type) are recipients successfully affected to change their attitude or behavior, thus increasing the effectiveness of the campaign. Two trends prevail in studies on this issue: The first proposes a linear dependence (the higher the intensity of negative emotions, the more effective the message). The second suggests the existence of a curvilinear relationship between intensity and effectiveness [17].

Generally, the effectiveness of negative appeals has been proved in many studies [32–41]. However, in the majority of studies, two levels of negative emotions were considered at most. Therefore, their results do not allow for unambiguous verification of examined relations (curvilinear and linear). An appropriate test for these models requires negative emotion to be manipulated using at least three levels within the same study to ensure that there is an intermediate level between the extremes [42].

Although research testing both models in the context of negative appeals has already been conducted in previous studies, it produced various and inconclusive results. Several studies have found that the higher level of negative emotions, the greater the effectiveness of ad [12, 20, 21, 43–47]. Other studies have found that moderate negative appeals perform better, producing an inverted-U-shaped model [18, 19, 22, 48–51]. The other studies stated that there is no clear evidence to support any of these models [42, 52–54].

One of the possible explanations for this discrepancy between outcomes is the fact that results reported in these publications are based mostly on declarations: questionnaires and focus groups that were used to determine both the emotions induced and the persuasive effect of the stimuli on participants. These methods do not allow for a comprehensive assessment of the ad’s effectiveness, because they are too rational and verbal [55] and are altered by cognitive processes [56].

Nowadays, thanks to advances in neuroscientific methods, we know that emotions can appear earlier than conscious processes and independently of them [57]. This means that an effective reaction to stimuli may arise beyond any conscious processing. The so-called “dual attitudes” [58] and “implicit attitudes” [6] assume that the same person can have two different attitudes toward an object at the same time: a conscious and an unconscious attitude. Thus, the natural consequence of this is the commonly observed dissociation between explicitly self-reported attitudes and implicit attitudes. Moreover, declarative measures are limited in their ability to predict advertising effectiveness, because of their limited ability to capture moment-by-moment cognitive processes (e.g. changes in attention, defense mechanisms), which may underlie the relationship between emotional reactions to ads and declarations [59].

Therefore, in the current study, we used both self-assessment measures and implicit neural reactions (measured by electroencephalography) to achieve a broader understanding of the processes that lead to effectiveness (or the lack of it) of social campaigns based on negative emotions.

## Neuroscientific methods in research on the effectiveness of social campaigns

Different aspects of social advertising have been already researched with the use of cognitive neuroscience tools [60–63]. The effectiveness of negative appeals has been studied as well. For this purpose, different tools have been used: skin conductance and heart rate [64, 65], electromyography [2, 66], facial expressions analysis [67, 68], eye-tracking [65, 69] and electroencephalography (event-related potentials) [23, 70]. In most cases, psychophysiological measures were used in addition to questionnaires [2, 64, 66, 67] or interviews [68]. Their purpose was to analyze behavioral changes, to assess and recognize emotional responses, and to predict the effectiveness of social ads. The use of neural measurements confirmed, for example, the existence of an attention disengagement process and avoidance responses that can occur during watching social advertising [23, 70].

In terms of the effectiveness of negative appeals, results are inconclusive as some experiments have shown that higher intensity of negative emotions used in social advertising has greater beneficial effects on cognition and behaviors [64, 66, 67, 71], while others have shown that the more negative the message is, the more avoidance responses and defense mechanisms are triggered [23, 69, 70]. The inconclusive nature of these results could be due to the fact that previous studies were usually testing only two different levels (low vs. high) of negative emotions intensity, what didn’t allow to check hypothesis about curvilinear relation between the intensity of negative emotions and the effectiveness of social advertising. Moreover, till now, researches testing relation between negative emotions appeal and social advertising effectiveness were based mostly on respondents’ declarations. In our study we included EEG measure detecting their unconscious and automatic emotional reactions to the ad.

## Brain oscillations as a marker of neural reactions to social campaigns

In our research, we wanted to focus on two neural measures of reactions to a social campaign: 1) frontal alpha asymmetry, and 2) beta oscillations. Frontal alpha asymmetry is the difference between the power of alpha waves in the left and right frontal hemispheres of the brain (e.g. [72, 73]). Frontal alpha asymmetry is thought to reflect the approach-withdrawal continuum—higher alpha power on the left hemisphere is related to a motivation to withdraw and reflects negative attitudes toward a given stimulus; conversely, higher alpha power on the right hemisphere is related to a motivation to approach and produce more positive attitudes [74]. In other words, the frontal alpha asymmetry is meant to differentiate between pleasant or engaging stimuli and unpleasant or disengaging ones. The application of the frontal alpha asymmetry index to the advertising materials has been already reported in numerous studies (e.g. [75–78]). Also, in the context of social campaigns (concerning anti-smoking announcements), Cartocci et al. [79] have shown—to some extent—a positive relationship between campaign effectiveness and approach motivation indicated by the frontal asymmetry among smokers.

As a second marker of neural reaction toward an ad, we chose to use beta oscillations. Research has shown that these types of oscillations are associated with reward processing [80–82] and integrating systems involved in attention and cognitive control [83–85]. In the context of advertising, an influential study by Boksem and Smidts [76] showed that beta oscillations are related to individual preference: the higher the amplitude of EEG beta oscillations when viewing a movie trailer, the higher the participants ranked a particular advertisement relative to the other movies viewed. Importantly, it was shown that beta oscillations captured unique information regarding individual preferences, not explained by declarative measures.

Based on the above research, we conclude that frontal alpha asymmetry (as a marker of withdrawal motivation) and beta oscillations (as a marker of attentive processing of the campaign message) are useful markers of implicit attitudes toward social campaigns, as they add more explanatory power than the use of declarative measures only. Frontal alpha asymmetry and beta oscillations could also be related (to some extent) to declarative emotions; however, it is assumed that this relationship is moderated by individual driving style.

## Driving style as a moderator of the relationship between reactions toward the message and intentions of behavioral change

An important and often neglected factor that may influence the persuasive mechanism of negative appeals are the individual differences related to the assessment of risks associated with dangerous behavior, such as speeding [86]. Many of the negative appeals models used in previous research ignored the role of these differences in the evaluation of social advertising effectiveness, which may also explain, to a large extent the different results of the research conducted in this area so far. Existing studies confirmed that individual experience, certain habits or perceived behavioral control can influence attitudes toward appeals for more healthy or safe behavior [87, 88].

Perceived self-efficacy in dealing with risk enhances the probability of non-adaptive reactions to the message of a social campaign, unrelated to the attempt to reduce the threat presented in the message, but rather to the attempt to diminish the negative emotional arousal evoked by the threatening content. These reactions may involve defensive avoiding, ignoring the message of a campaign or rejecting its personal relevance [89]. The efficient use of emotion regulation strategies can help to avoid considering possible alternative behavior or processing the information in an unbiased manner (see [90–92]). The existence of these defense mechanisms was confirmed by Kessels et al. [23, 70] who used EEG to measure the level of attention directed toward a threatening message by individuals at the greatest risk (in this case: smokers vs. non-smokers). They showed that being in a risk group may lead to a decrease in the attentional resources dedicated to the processing of negative appeals.

On the basis of the above research, we assumed that including measurements of individuals’ driving styles was important to understanding the complex relationship between negative emotional appeals and the intentions of behavioral change. We hypothesized that drivers who prefer risky driving styles would exhibit different patterns of relationships between neural reactions to social campaign and declarative measurements of negative emotions due to emotion regulation strategies aimed at reducing the threat (defense mechanisms). As the current study shows, effective application of these strategies is related to a decreased intention to change behavior—in this context, to drive more safely.

## Overview of the research

The effectiveness of negative emotional appeals in social campaigns has been the subject of numerous studies, but their results are not fully conclusive. In order to examine this issue in a comprehensive way, we integrated three key aspects of negative emotional appeals into a single study. First, the use of three different levels of negative emotions in a social campaign allowed us to test both linear and curvilinear hypotheses about the impact of emotions on behavioral intentions. Second, we employed two different types of measures: self-report questionnaires and brain reactions measured with EEG in order to examine explicit and implicit reactions toward the social campaign. Third, we considered individual differences in behavioral style—measuring the driving style of the participants will allow us to identify potential differences in the processing of threatening information by people who are safe or risky drivers.

**Fig 1:**
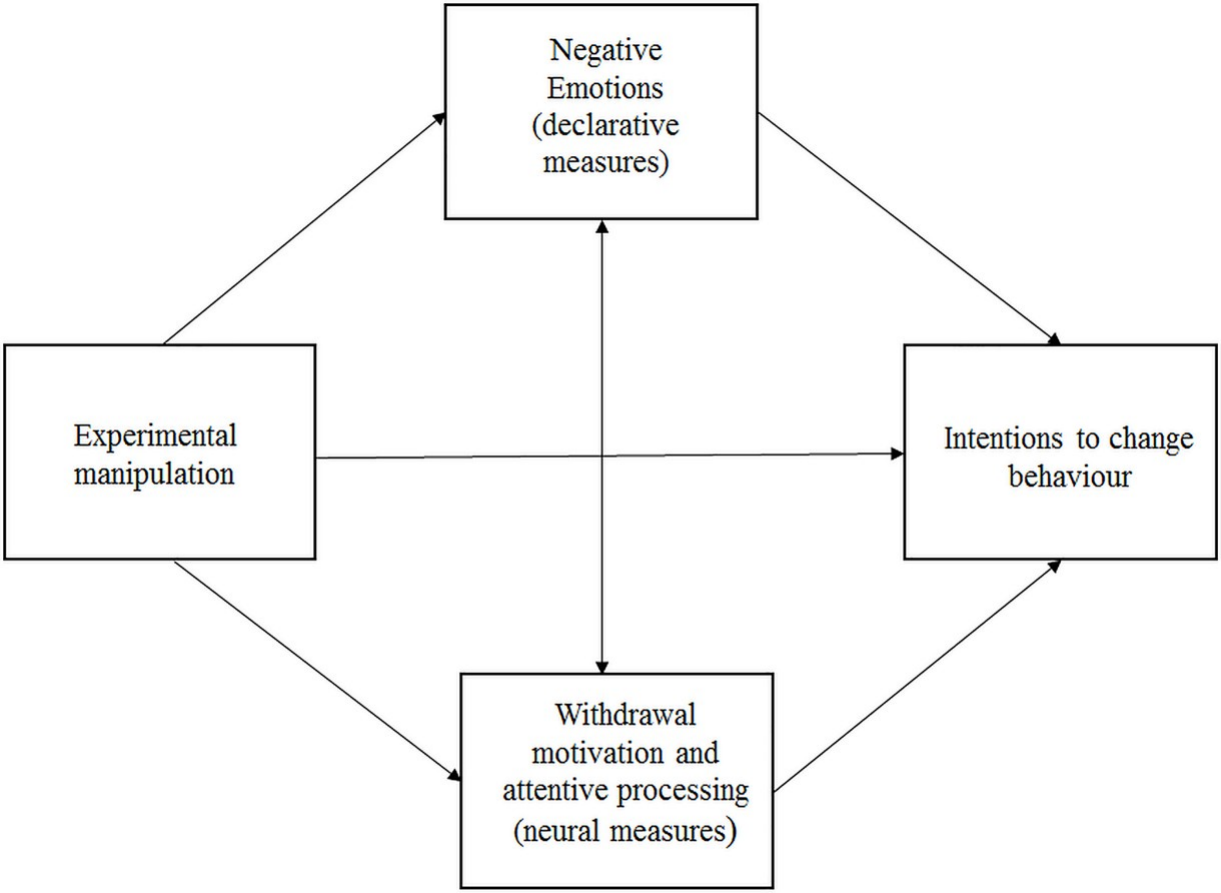
Theoretical model of the relationship between experimental manipulation and intention to change behavior via declarative and neural attitudes toward a social campaign.

Our analytical plan consisted of two steps. First, we planned to examine the main effects and interaction of experimental manipulation and driving style on dependent variables. Then, we wanted to examine linear vs. curvilinear hypotheses on the relationship between the intensity of negative emotions in social campaigns and intention to change behavior (in our context, less risky driving). We assumed that the social campaign would influence these intentions by increasing the negative emotions felt by the participant (declarative measures) and would change the participant’s motivation, attention and reward processing (neural measures). To confirm this assumption, we used a mediational analysis (Fig 1).

**Fig 2:**
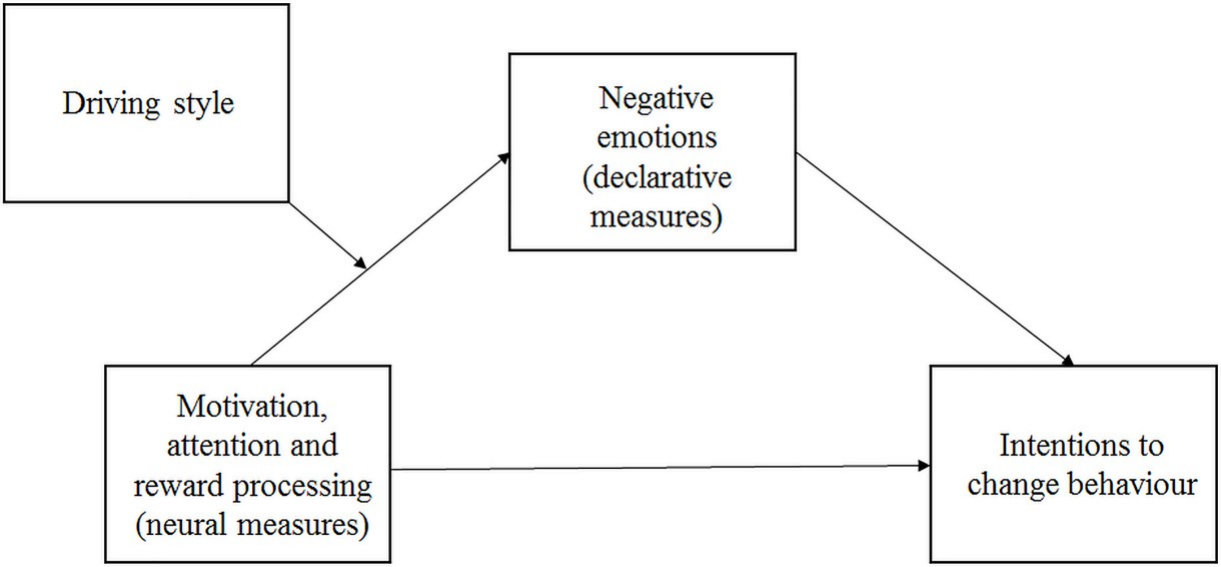
Theoretical model of moderated mediation.

Second, we assumed that social advertising promoting safe driving that is using negative emotions will be less effective in case of risky drivers than non-risky drivers. In case of risky drivers, intense negative emotions used in social ads will be more threatening and therefore by evoking defense mechanisms, will lower ad effectiveness. Additionally, we examined whether this effect was dependent or independent of experimental conditions. To do so, we used a moderated mediation analysis (Fig 2).

## Methods

### Participants

The study was conducted with students and employees of three different Polish universities: the University of Szczecin, the West Pomeranian University of Technology Szczecin and the Maritime University of Szczecin. For the experiment, 64 persons (32 men) were recruited with the use of an online questionnaire. Their mean age was 38±13.

### Stimuli

**Fig 3:**
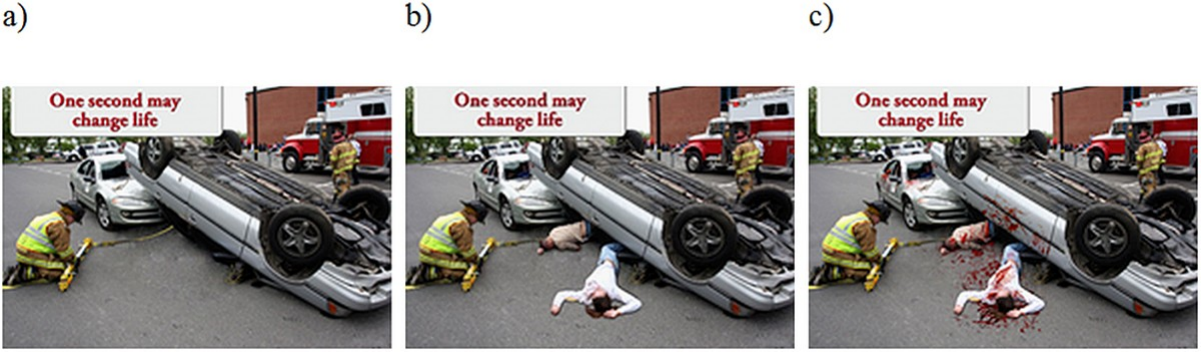
Stimuli used in the experiment: a) low intensity of negative emotions, b) medium intensity of negative emotions, c) high intensity of negative emotions. Reprinted from [95] under a CC BY license, with permission from Alamy, original copyright 2009.

For the experiment, we used advertising related to a road safety promotion. The printed social advertising was created using a stock photo showing a staged car accident. The original image was modified to obtain three different levels of negative emotional intensity. Modifications that were performed included altering the victims’ presentation and adding a campaign slogan announcing that “One second can change your life” (Fig 3). Before using the images in this experiment, graphic materials were pre-tested to determine whether they had been prepared in a proper manner and whether the intensity of the negative emotions was appropriately graded [93]. Respondents (86 female and 74 male) assessed the stimuli in terms of three basic negative emotions [94]–fear, disgust and sadness. They done it on a scale from 1 to 7 (from minimal to maximum level of sensations). Each of the basic negative emotions was assessed independently. In order to determine the type and level of emotions evoked by prepared research materials, the three versions of advertising were tested by independent group of respondents (between subject experimental design) and evaluated on the same scale using the same questions. Conducted analyses (ANOVA and t-tests) allowed to determine that there are differences of averaged intensity of emotion felt between groups. The results confirmed that the images show various levels of negative emotions–from the lowest to the highest, so we can assume that the manipulation was valid.

### Procedure

The experiment involved the presentation of the stimuli in a laboratory environment with the use of cognitive neuroscience tools. At this stage, participants were offered an incentive for participation (a portable battery pack). The study was approved by the local ethical committee (Bioethical Commission of Regional Chamber of Physicians and Dentists in Szczecin; approval number: 12/KB/V/2013) and written informed consent was obtained from each subject after explanation of the course of study.

Experimental stimuli were displayed using the following procedure. A black image with a rotating triangle displayed for 1 minute was used to focus the participant’s attention on the screen and to record a baseline activity for psychophysiological signals. A reference image was taken from the International Affective Picture System (IAPS) database [96] (image number 8030) as it was assessed as inducing high arousal. This image was used to familiarize the participants with the method of presenting the stimuli and operating in the experimental environment. After that, the main stimulus was shown. Each participant saw one of the three versions of the social advertisement (between subject design). The image was displayed for 30 seconds. This allowed participants the chance to look at all details of the presented social advertisement. After the ad exposure, participants were asked a set of questions about the ad, the campaign and their attitudes toward the advertised issue. Time for answering questions was not limited—every person could answer them at her or his own pace.

### Psychophysiological data recording and processing

The brain activity was recorded using a g.Nautilus device (sampling rate of 500 Hz). For the needs of this research, electrodes were installed on the scalp in compliance with the guidelines of the 10–20 system [97]. The EEG signals were bandpass filtered to remove data with frequencies below 0.4 Hz and above 50 Hz [98]. Then, artifacts were removed with the use of a blind source separation (BSS) technique.

The main method which was applied was wavelet independent component analysis (WICA) [99], which has been proven efficient at detecting and removing artifacts in EEG recordings, both in the time and frequency domains. By using discrete a wavelet transformation (DWT) function, the signal for each channel was decomposed into five different frequency bands (alpha, theta, beta, gamma and delta) depending on Daubechies 8 wavelet function [100]. These EEG traces were then segmented to obtain cerebral activity during the observation of the social advertisement and the black screen.

Due to technical issues, recordings of psychophysiological signals for two subjects were not successful, and data gathered from this participant was excluded from further analysis. One participant was excluded due to an outlying frontal asymmetry value (Z> 3.0) (see also [101]).

### Measures

Driving style of the participants was assessed on the basis of the Driver Behavior Questionnaire (DBQ) before the main experiment. The survey uses questions from a study by Reason and others [102] with further modifications [103, 104]. For the purpose of this research, two factors measured by the DBQ were taken into account: conscious violations and tactical mistakes of drivers. Altogether, the questionnaire consisted of 16 statements, and participants of the study were asked to assess how often they commit each of the errors (e.g. overtaking a vehicle that is slowing down or misjudging speed at a main road exit). For the assessment, a Likert response-scale was used, where 0 = *never* and 5 = *very often*.

The questionnaire was conducted in order to divide the respondents into two groups: safe and risky drivers. This division was made based on the sum of all answers given for the DBQ questions. After collecting all responses, the median value of the answers’ sum was calculated—separately for men and women. This was done because men had a higher tendency to commit driving errors, thus achieving greater values in the questionnaire. The median for women in the surveyed group was 21, and for men, 25.5. All subject of both genders who achieved the result lower or equal to median were classified as safe drivers; the rest were classified as risky drivers.

During the main experiment, we used two different types of measurement: self-report measurements collected using questionnaires and psychophysiological measures based on EEG data. Questionnaires that participants were asked to complete contained three different measures: (a) emotions evoked by the ad, (b) advertising evaluation and (c) intention to change behavior.

A list of emotions was taken into account—which included anger, wrath, torment, disgust, sadness, fear and terror—to represent the basic categories of negative emotions [105]. Such emotions were chosen in accordance with the stimuli character. The question for each item was, “To what extent, by looking at the previously displayed image, do you feel following emotion?” Answers were measured on a scale from 1 = *not at all* to *7 = very strongly*. All answers were averaged in order to create a *negative emotion index*: α = .91.

*Advertising evaluation* was measured using three questions: “Do you think the idea of this social advertising is right?” “Do you think that the presented social advertising is prepared in a professional manner?” and “Do you think that the slogan matches this social advertising?” The answers were measured on a scale from *1 = not at all* to *7 = very strongly*.

*Expected ad effectiveness* was measured with one item: “What do you think, to what extent the advertisement will cause you to drive more carefully?” The answers were measured on a scale from *1 = not at all* to *7 = very strongly*.

The EEG data was used to calculate measures that assessed the effectiveness of social advertising on implicit reactions and cognitive processes. We used frontal alpha asymmetry as a marker of withdrawal motivation. This is calculated using a signal registered by electrodes Fp1, F7, F3, Fp2, F8, F4, according to the following formula [106, 107]:

$$\begin{array}{c} WM=\frac{1}{N_{P}}\sum_{i\in P}x_{\alpha_{i}}^{2}(t)-\frac{1}{N_{Q}}\sum_{i\in Q}y_{\alpha_{i}}^{2}(t)=\\ =Average\;Power_{\alpha_{right,frontal}}-Average\;Power_{\alpha_{left,frontal}} \end{array}$$ (1)

where:

$x_{\alpha_{i}}$ and $y_{\alpha_{i}}$ – *i*-th EEG channel in the alpha band (right and left frontal lobes, respectively),

P and Q–sets of right and left channels,

N_P_ and N_Q_−cardinality of P and Q.

We also calculated beta oscillations as a marker of attentive processing of stimuli from electrodes Fz, F4 and F4.

## Results

First, we examined the correlations between EEG measures (frontal alpha asymmetry as a marker of withdrawal motivation and beta oscillations as a marker of attentive processing) and three levels of advertising effectiveness (based on declarations): negative emotion index, intention to change behavior and campaign evaluation. The negative emotion index was positively correlated with the expected campaign effectiveness and campaign evaluation. Neither frontal alpha asymmetry nor beta oscillations were significantly correlated with declarative measures. However, they were negatively correlated with each other, which underlines their opposite, to some extent, meaning on the neural level: withdrawal vs. attentive processing of the message.

All zero-order correlations between the dependent variables are presented in Table 1.

**Table 1:** Means, standard deviations, and correlations between dependent variables.

Variable	M	SD	1	2	3	4
1. Negative emotion index (NEI)	3.07	1.53				
2. Intention to change behavior (ICB)	3.79	1.62	.52**			
3. Campaign evaluation (CE)	4.70	1.30	.27*	.48**		
4. Frontal alpha asymmetry	.003	.47	-.014	.22	-.06	
5. Beta Oscillations	-.02	.33	-.20	-.12	.004	-.27*

* *p* < .05

^**^ *p* < .001

In order to analyze the effects of experimental manipulation and driving style, we conducted two multivariate analyses of variance (MANOVAs) on declarative and neural dependent variables separately [108]. First, we analyzed the effects of experimental manipulation and driving style on declarative measures. We conducted a 2x3 MANOVA with driving style (risky vs. safe) and intensity of negative stimuli in a social campaign (low, medium or high–experimental manipulation) as between subject factors and the negative emotion index (NEI), intention to change behavior (ICB), and campaign evaluation (CE) as dependent measures.

The analysis revealed a significant multivariate effect of the manipulation, Wilks’s Lambda = .70, F(6, 106) = 3.46, p = .004, ηp2 = .16. However, there were neither significant effects of driving style (Wilk’s lambda = .97, F(3,53) = .55, p = .65, ηp2 = .03) nor interaction between manipulation and driving style (Wilk’s lambda = .94, F(6,106) = .59, p = .73, ηp2 = .03).

The univariate effect of the manipulation was significant for the NEI, F(2,55) = 5.33, p = .008, ηp2 = .16. Participants in the high emotional intensity condition felt more negative emotions (M = 3.77, SD = 1.67) than participants in the low intensity condition (M = 2.35, SD = 1.22), p = .006, but similar to those in the medium condition (M = 3.20, SD = 1.35), p = .73. There was no significant difference between the low and medium intensity conditions (p = .20). We also did not observe significant effects of manipulation on either the intention to change behavior (ICB) or campaign evaluation (CE).

**Fig 4:**
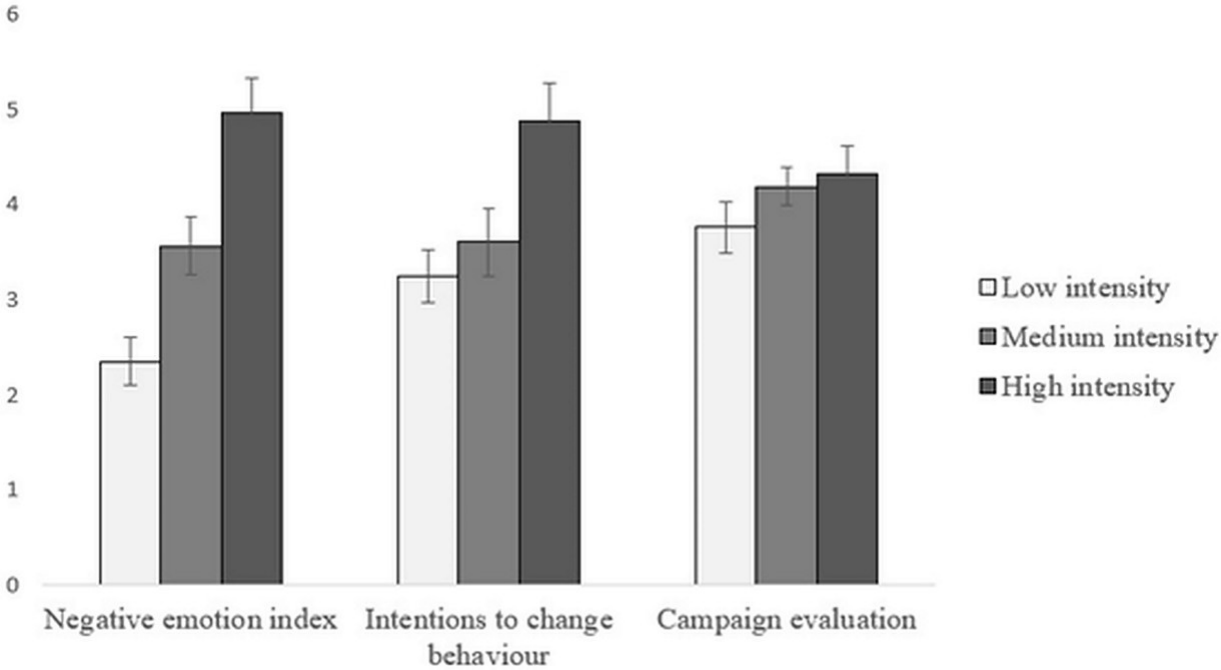
Negative emotions felt by participants, intention to change behavior, and campaign evaluation as a function of the intensity of negative emotions in the social campaign. Error bars represent ±1 standard errors.

**Fig 5:**
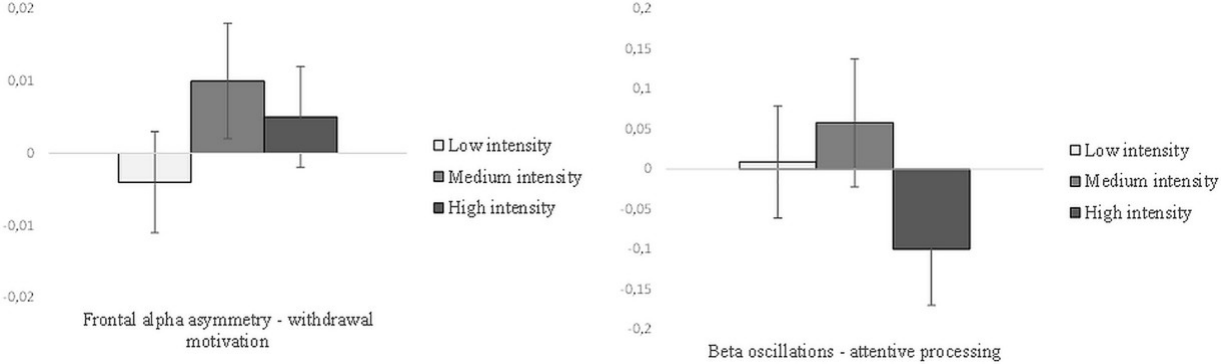
Frontal alpha asymmetry and beta oscillations as a function of the intensity of negative emotions in the social campaign. Error bars represent ±1 standard errors.

In the second 2x3 MANOVA, we examined the effect of the same independent variables: driving style and experimental manipulation (and their interaction) as between subject factors and on both neural measures: frontal alpha asymmetry and beta oscillations as dependent variables. The analysis did not reveal a significant multivariate effect for neither of the experimental manipulation (Wilks’s lambda = .92, F(4, 108) = 1.10, p = .36, ηp2 = .04), driving style (Wilk’s lambda = .99, F(2,54) = .15, p = .86, ηp2 = .01) nor interaction between manipulation and driving style interaction (Wilk's lambda = .97, F(4,108) = .47, p = .75, ηp2 = .02). The results of both MANOVAs are shown in Figs 4 and 5.

**Fig 6:**
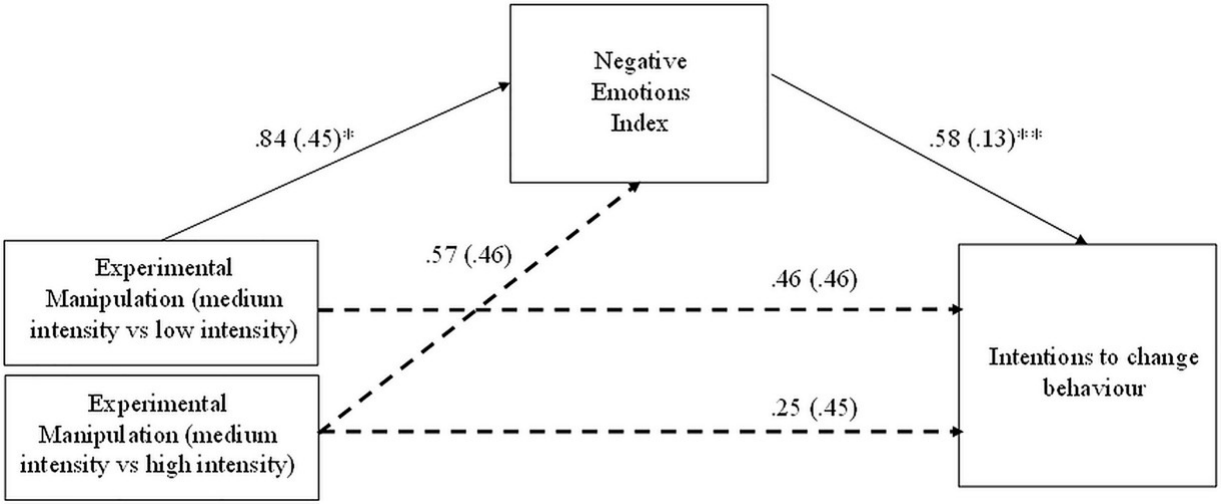
Indirect effect (IE) of experimental manipulation on the intention to change behavior via negative emotions evoked by the social campaign.

Because the experimental manipulation did not influence neural measures, we decided to include only the declarative measurement of negative emotions in the planned mediation analysis presented in Fig 1. The aim of this analysis was to examine whether experimental manipulation influences ICB via increased negative emotions. We conducted an indirect effect (IE) analysis using macro PROCESS v. 2.16.3 for SPSS (model 4, 10 000 samples bootstrapping, [109]). The analysis revealed that the negative emotion index (NEI) mediated between the contrast in the low intensity condition vs. medium intensity condition and ICB, IE = .49, SE = .28, CI95% [.025; 1.13], and low intensity condition vs. high intensity condition and ICB, IE = .82, SE = .29, CI95% [.29; 1.46]. Importantly, there was no significant indirect effect of the NEI on ICB in the medium intensity condition compared to the high intensity condition (IE = .33, SE = .28, CI95% [- .26; .88]). This result suggests that above a certain level of emotional intensity (in this case, medium), increasing negative emotions via more shocking scenes does not translate into an intention to driving in a safer manner (Fig 6).

Finally, we examined whether driving style moderates the relationship between neural reactions toward the social campaign and declarative measures of negative emotions felt by the participants and whether these emotions translate into lower ICB (see Fig 2). We assumed that the intensity of neural reactions among risky drivers would correlate with lower declarative emotions due to the appearance of effective defense mechanisms. As the MANOVAs did not yield a significant effect of experimental manipulations on neural measures but our experimental manipulation significantly influenced the level of declarative emotions, we used experimental conditions as control variables in moderated mediation analysis.

We tested two moderated mediation models (macro PROCESS, model 7, 10 000 samples bootstrapping) for frontal alpha asymmetry and beta oscillations separately. In the case of the frontal alpha asymmetry, its interaction with driving style was not significant, B = -.37, SE = .80, p = .56, CI95% [-.1.98; 1.23]. So, we did not observe a significant indirect effect of frontal alpha asymmetry on ICB via the NEI among risky (IE = -.06, SE = .33, CI95% [-.68; .66]) or safe drivers (IE = -.28, SE = .43, CI95% [-1.32; .37]).

**Fig 7:**
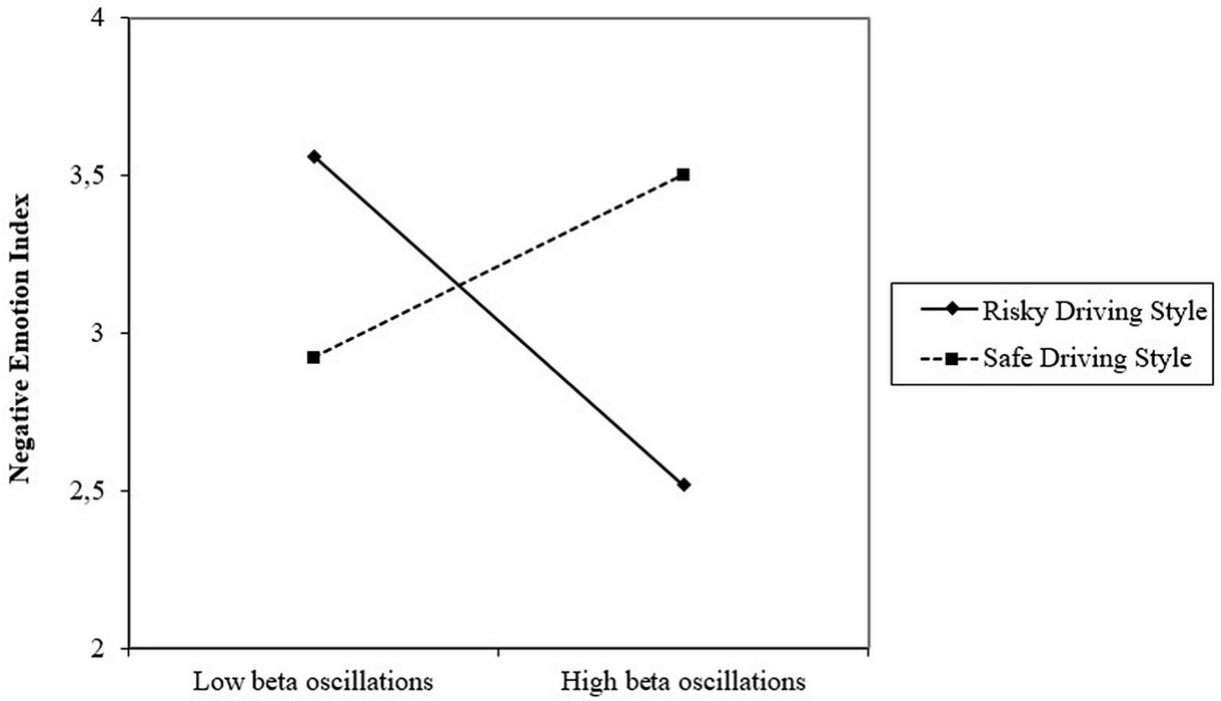
Moderating effect of driving style on the relationship between beta oscillations and the NEI (declarative measure).

In turn, interaction between beta oscillations and driving style yielded significant (on the trend level) results: B = 2.22, SE = 1.16, p = .06, CI95% [-.13; 4.55]. We observed a significant conditional effect of beta oscillations on the NEI among risky drivers: B = -1.38, SE = .65, p = .04, CI95% [-2.71; -.07]. Among safe drivers, the conditional effect was not significant: B = .82, SE = .96, p = .39, CI95% [-1.10; 2.75] (Fig 7). This effect confirmed, to some extent, our hypothesis on different cognitive processes employed by risky drivers when viewing a threatening message in comparison to safe drivers. In the case of more risky drivers, the attention they paid to threatening messages was related to the negative emotions they felt. The more they paid attention (increased beta oscillations) to threatening messages, the fewer negative emotions they declared, which could be the effect of different defense mechanisms or emotion regulation strategies used by risky drivers.

However, the full model of moderated mediation was not significant (index of moderated mediation = 1.26, SE = .93, CI95% [-.32; 3.36]).

## Discussion

The aim of our research was to examine which of two competing models, linear or curvilinear, is better at explaining the relationship between negative emotional appeals and the effectiveness of social campaigns. Specifically, we wanted to examine if and how negative emotions evoked by a social campaign with low-, medium- and high-intensity levels translate into intentions to driving more safely. Taking into account the limitations of previous studies, we used both declarative and neural measures in this study to check explicit and implicit reactions to a campaign. Additionally, we examined whether individual differences in driving style are related to the effectiveness of the campaign and specific processing of negative emotional appeals by risky and safe drivers.

In line with Albouy’s [9] study, we observed a linear increase in negative emotions as a function of the emotional intensity of the social campaign. This result is also consistent with a meta-analysis, including 13 experimental studies, which showed that threat appeals are an effective way of evoking negative emotions among drivers [110]. However, the main aim of a successful social campaign is not to arouse negative emotions in the audience, but to change attitudes or behavior in a prosocial way. By using indirect effect analysis, we showed that the increase in negative emotions felt by participants translates into intentions to drive safer only to a certain—medium—level of negative emotion in a campaign. Further increase of negative emotions was not associated with an enhanced intention to commit less risky behavior. Therefore, the results of our study suggest that the curvilinear hypothesis is more accurate than the linear in the context of effectiveness of negative emotional appeals—despite the fact that we did observe significantly lower intension to change behavior (ICB) in the most shocking condition, we also did not observe any improvement.

Our results show that effective campaign communication does not have to use the particularly shocking content to evoke positive changes in attitudes. This result is particularly important in the context of the serious ethical concerns about the use of negative emotional appeals (for the review, see, e.g. [111]).

An important issue to take into account before the potential generalization of our results is their specific context–a warning against a particularly risky behavior. The message of the advertisement was focused on changing an individual’s habits in order to protect him or her from danger. When campaign communication targets an individual’s feeling of safety, he or she could use different defense mechanisms than in reaction to a campaign for the benefit of others. Indeed, research on charitable giving, for example, has shown a linear relationship between the number of negative emotions felt by participants and their intention to respond favorably or donate money [9] (see also [112]). Therefore, subsequent studies might examine the relationship between negative emotional appeals and intension to change behavior (ICB) in situations when the campaign message addresses the safety of others, such as co-passengers or other road users.

Contrary to expectations, we didn’t observed differences in neural reactions to the campaign depending on the manipulation (intensity of the negative emotions in the advertising message): frontal alpha asymmetry (as a marker of withdrawal motivation) and beta oscillations (as a marker of attentive processing of the message). Moreover, we did not observe a significant relationship between neural measures and declared negative emotions. Thus, we decided not to include neural variables in our indirect effect model.

There are several reasons why in our study neural indicators were not associated with our dependent variable and declarative negative emotions. First, we must consider that neural and declarative measurements are capturing different area and therefore they are often not related to each other [113]. Moreover, there are proves that variables measured in the same way will be more strongly correlated with one another. A similar effect was found by Hakim et al. [114], who, in the context of commercial advertising, noted that the questionnaire measures used in their survey were better predictors of other questionnaires than neural indicators. Second, it is highly possible that neural measures are better predictors of actual behavioral change than declarations are. For example, Tullet, Harmon-Jones and Inzlicht [101] argued that frontal alpha asymmetry might be related to a behavioral measure of prosociality, although it is not related to the declarative measures of helping intentions. Lack of measurement of behavioral change was, therefore, a major limitation of our study. In order to understand the role of brain oscillations in processing a social campaign message, it would be worth including this type of measure in subsequent experiments, e.g. by using a driving simulator or gathering data of participants’ average driving speeds at a specific time after the experiment.

Third, previous research on the relationship between brain oscillations and reactions to advertisements was mainly limited to commercial advertising (e.g. [75, 76, 114]). Social campaigns, by their very nature, engage the viewer in a different way and may cause different, possibly more complicated, reactions. First of all, social advertising usually requires a deeper change of behavior than commercial. Moreover, social ads, by frequent use of negative emotions, can often evoke negative and unpleasant viewer reactions; therefore, the chance of raising defense mechanisms is greater. As a consequence, findings that identify how commercial advertising works are not always transferred to social ads.

Additionally, in analyzing the moderated mediation, we examined whether the relationship between brain reactions and declarative measure of negative emotions is different depending on the driving style and whether these differences influenced behavioral intension (declaration to change own driving style). The results reveal that in the group of risky drivers, the number of beta oscillations was negatively related to declared negative emotions. Such a relationship was not observed among safe drivers. This result suggests that risky drivers who pay attention to the negative message could effectively use defensive mechanisms in order to prevent negative emotions. Although these results need to be interpreted with some caution, as the moderation effect proved to be only marginally significant, they may indicate that the risky drivers who paid more attention to the campaign also applied efficient defense strategies in order to reduce emotional discomfort. This result was observed when controlling for the influence of experimental conditions and can, therefore, be considered a general mechanism independent of the intensity of negative emotional appeals. The observed relationship broadens the existing findings on the use of defense mechanisms by the recipients from the risk group in response to the threatening message [23, 70]. Using an evoked potential analysis method, Kessels et al. [70] discovered that participants from the risk group (in this case, smokers) employed different strategies in response to a campaign. Specifically, they engaged fewer attentional resources to process the presented content. In turn, our results may provide preliminary evidence that even engaging in message processing reduces the negative emotions that risky drivers feel, rather than raising them. However, the analysis of moderated mediation did not show that the above mechanism decreased ICB among risky drivers (the index of moderated mediation was not significant).

Further research should also determine which specific defense mechanisms are used by the risk group to reduce emotional discomfort and how these mechanisms are related to changes in brain oscillations. A key aspect of this research will be to examine the precise role of beta oscillations in the use of defense mechanisms, e.g. whether they are (1) definitely associated with more attentive message processing, which is a prerequisite for the implementation of defense mechanisms, or rather (2) a marker of implementing emotional regulation *per se* (as some studies have suggested beta oscillations are involved in the process of top-down cognitive control, e.g. [83, 115]).

Despite the above limitations, we believe that the results of the presented study are an important contribution to the field of research on the effectiveness of negative emotional appeals and are also an inspiration for further research to extend our findings. First, we found that using strong negative emotions in a social campaign has limited practical sense, as it does not translate into ICB. Second, we found that beta oscillations could be related to the use of maladaptive defense mechanisms during campaign message processing among risk groups.

## Supporting information

S1 File Data used in statistical analysis. (SAV)

## References

[1] Donovan R, Henley N. Social marketing. Melbourne, Australia: IP Communications; 2003.

[2] Missaglia A, Oppo A, Mauri M, Ghiringhelli B, Ciceri A, Russo V. The impact of emotions on recall: An empirical study on social ads. Journal of Consumer Behaviour. 2017;16(5):424–433.

[3] Grigoryan N. Ethics of a Social Marketing Campaign: An Integrative Assessment Model. Journal of Media Ethics. 2019;34(2):114–127.

[4] Costarelli S, Colloca P. The effects of attitudinal ambivalence on pro-environmental behavioural intentions. J Environ Psychol. 2004;24(3):279–288.

[5] Moser A. Thinking green, buying green? Drivers of pro-environmental purchasing behavior. J Consum Mark. 2015;32(3):167–175.

[6] Greenwald A, Banaji M. Implicit social cognition: Attitudes, self-esteem, and stereotypes. Psychol Rev. 1995;102(1):4–27. 10.1037/0033-295x.102.1.4 7878162

[7] Rotfeld JH. Understanding advertising clutter and the real solution to declining audience attention to mass media commercial messages. J Consum Mark. 2006;23(4):180–181.

[8] Lee S, Cho Y. Do Web Users Care About Banner Ads Anymore? The Effects of Frequency and Clutter in Web Advertising. Journal of Promotion Management. 2010;16(3):288–302.

[9] Albouy J. Emotions and prosocial behaviours: A study of the effectiveness of shocking charity campaigns. Recherche et Applications en Marketing (English Edition). 2017;32(2):4–25.

[10] Chang C, Lee Y. Framing Charity Advertising: Influences of Message Framing, Image Valence, and Temporal Framing on a Charitable Appeal1. J Appl Soc Psychol. 2009;39(12):2910–2935.

[11] Maison D, Pawłowska B. Using the Facereader Method to Detect Emotional Reaction to Controversial Advertising Referring to Sexuality and Homosexuality In: Nermend K, Łatuszyńska M, ed. by. Neuroeconomic and Behavioral Aspects of Decision Making. Cham: Springer; 2017 p. 309–327.

[12] Witte K, Allen M. A Meta-Analysis of Fear Appeals: Implications for Effective Public Health Campaigns. Health Educ Behav. 2000;27(5):591–615. 10.1177/109019810002700506 11009129

[13] Nabi R. Emotional Flow in Persuasive Health Messages. Health Commun. 2014;30(2):114–124. 10.1080/10410236.2014.974129 25470436

[14] Chen M. Impact of fear appeals on pro-environmental behavior and crucial determinants. Int J Advert. 2015;35(1):74–92.

[15] Gallopel K, Valette-Florence P. Fear appeals in anti-tobacco campaigns: cultural considerations, the role of fear, proposal for an action plan. Valdosta: Association for Consumer Research; 2002 p. 274–279.

[16] Terblanche-Smit M, Terblanche N. HIV/Aids marketing communication and the role of fear, efficacy, and cultural characteristics in promoting social change. J Public Aff. 2011;11(4):279–286.

[17] Blondé J, Girandola F. When Defensive Reactions Contribute to the Acceptance of Fear-Arousing Communications. Curr Psychol. 2017;38(1):75–83.

[18] Keller P. Converting the unconverted: The effect of inclination and opportunity to discount health-related fear appeals. J Appl Psychol. 1999;84(3):403–415. 10.1037/0021-9010.84.3.403 10380420

[19] Roskos‐Ewoldsen D, Yu J, Rhodes N. Fear appeal messages affect accessibility of attitudes toward the threat and adaptive behaviors. Commun Monogr. 2004;71(1):49–69.

[20] Lennon R, Rentfro R. Are young adults fear appeal effectiveness ratings explained by fear arousal, perceived threat and perceived efficacy. Innovative Marketing. 2010;6(1):58–65.

[21] McCloud R, Okechukwu C, Sorensen G, Viswanath K. Cigarette graphic health warning labels and information avoidance among individuals from low socioeconomic position in the U.S. Cancer Causes Control. 2017;28(4):351–360. 10.1007/s10552-017-0875-1 28255678

[22] Janis IL. Effects of fear arousal on attitude change: Recent developments in theory and experimental research In: Berkowitz L, ed. by. Advances in Experimental Social Psychology, 3 Burlington: Elsevier; 1967.

[23] Kessels L, Ruiter R, Jansma B. Increased attention but more efficient disengagement: Neuroscientific evidence for defensive processing of threatening health information. Health Psychol. 2010;29(4):346–354. 10.1037/a0019372 20658820

[24] Blumberg S. Guarding against Threatening HIV Prevention Messages: An Information-Processing Model. Health Educ Behav. 2000;27(6):780–795. 10.1177/109019810002700611 11104375

[25] Bakalash T, Riemer H. Exploring ad-elicited emotional arousal and memory for the ad using fMRI. J Advert. 2013;42(4): 275–291.

[26] Chen P, Chen F, Zhang L, Ma X, Pan X. Examining the influence of decorated sidewaall in road tunnels using fMRI technology. Tunn Undergr Sp Tech. 2020;99:103362.

[27] Sharma P, Gountas J, Gountas S, Ciorciari J. Looking beyond traditional measures of advertising impact: Using neuroscientific methods to evaluate social marketing messages. J Bus Res. 2019;97:1–45.

[28] Yang DJ. Exploratory Neural Reactions to Framed Advertisement Messages of Smoking Cessation. Soc Mar Q. 2018;24(3):216–232.

[29] Janis I, Feshbach S. Effects of fear-arousing communications. J Abnorm Soc Psychol. 1953;48(1):78–92. 10.1037/h0060732 13022199

[30] Grancea I. When Images Hurt: A Closer Look at the Role of Negatively-Valenced Images in Advertising. Argumentum. Journal the Seminar of Discursive Logic, Argumentation Theory & Rhetoric. 2012;10(2):153–164.

[31] McGraw A, Schiro J, Fernbach P. Not a Problem: A Downside of Humorous Appeals. Journal of Marketing Behavior 2015, 1(2): 187–208.

[32] Biener L, Ji M, Gilpin E, Albers A. The Impact of Emotional Tone, Message, and Broadcast Parameters in Youth Anti-smoking Advertisements. Journal of Health Commun. 2004;9(3):259–274. 10.1080/10810730490447084 15360037

[33] Passyn K, Sujan M. Self‐Accountability Emotions and Fear Appeals: Motivating Behavior. J Consum Res. 2006;32(4):583–589.

[34] Lewis I, Watson B, White K. An examination of message-relevant affect in road safety messages: Should road safety advertisements aim to make us feel good or bad?. Transp Res Part F Traffic Psychol Behav. 2008;11(6):403–417.

[35] Porzig-Drummond R, Stevenson R, Case T, Oaten M. Can the emotion of disgust be harnessed to promote hand hygiene? Experimental and field-based tests. Soc Sci Med. 2009;68(6):1006–1012. 10.1016/j.socscimed.2009.01.013 19181428

[36] Durkin S, Biener L, Wakefield M. Effects of Different Types of Antismoking Ads on Reducing Disparities in Smoking Cessation Among Socioeconomic Subgroups. Am J Public Health. 2009;99(12):2217–2223. 10.2105/AJPH.2009.161638 19833980 PMC2775761

[37] Charry K, Demoulin N. Behavioural evidence for the effectiveness of threat appeals in the promotion of healthy food to children. Int J Advert. 2012;31(4):773–794.

[38] Noble G, Pomering A, Johnson LW. Gender and message appeal: their influence in a pro-environmental social advertising context. J Soc Mark. 2014;4(1):4–21.

[39] Giachino C, Stupino M, Petrarulo G. The role of emotions in advertisement: a first investigation. In: Innovation, entrepreneurship and sustainable value chain in a dynamic environment. Euromed Press; 2015.

[40] Krishen A, Bui M. Fear advertisements: influencing consumers to make better health decisions. Int J Advert. 2015;34(3):533–548.

[41] Plant B, Irwin J, Chekaluk E. The effects of anti-speeding advertisements on the simulated driving behaviour of young drivers. Accid Anal Prev. 2017;100:65–74. 10.1016/j.aap.2017.01.003 28119216

[42] Tannenbaum M, Hepler J, Zimmerman R, Saul L, Jacobs S, Wilson K et al Appealing to fear: A meta-analysis of fear appeal effectiveness and theories. Psychol Bull. 2015;141(6):1178–1204. 10.1037/a0039729 26501228 PMC5789790

[43] Higbee K. Fifteen years of fear arousal: Research on threat appeals: 1953–1968. Psychol Bull. 1969;72(6):426–444. 10.1037/h0028430 4905677

[44] Leventhal H. Findings and theory in the study of fear communications In: Advances in experimental social psychology. New York: Academic Press; 1970.

[45] LaTour M, Pitts R. Using fear appeals for AIDS prevention in college aged population: An analysis of arousal and ad response. Journal of Healthcare Marketing. 1989;9:5–14. 10303935

[46] Donovan R, Jalleh G, Henley N. Executing effective road safety advertising: are big production budgets necessary?. Accid Anal Prev. 1999;31(3):243–252. 10.1016/s0001-4575(98)00074-8 10196601

[47] Delaney A, Lough B, Whelan M, Cameron M. A review of mass media campaigns in road safety. Victoria: Monash University Accident Research Centre Reports; 2004.

[48] Ray M, Wilkie W. Fear: The Potential of an Appeal Neglected by Marketing. J Mark. 1970;34(1):54.

[49] Krisher H, Darley S, Darley J. Fear-provoking recommendations, intentions to take preventive actions, and actual preventive actions. J Pers Soc Psychol. 1973;26(2):301–308. 10.1037/h0034465 4144987

[50] Kohn P, Goodstadt M, Cook G, Sheppard M, Chan G. Ineffectiveness of threat appeals about drinking and driving. Accid Anal Prev. 1982;14(6):457–464.

[51] Quinn V, Meenaghan T, Brannick T. Fear Appeals: Segmentation is the Way to Go. Int J Advert. 1992;11(4):355–366.

[52] Lang A, Yegiyan N. Understanding the Interactive Effects of Emotional Appeal and Claim Strength in Health Messages. J Broadcast Electron Media. 2008;52(3):432–447.

[53] Peters G, Ruiter R, Kok G. Threatening communication: a critical re-analysis and a revised meta-analytic test of fear appeal theory. Health Psychol Rev. 2013;7(sup1):S8–S31. 23772231 10.1080/17437199.2012.703527 PMC3678850

[54] Clayton R, Leshner G, Bolls P, Thorson E. Discard the Smoking Cues—Keep the Disgust: An Investigation of Tobacco Smokers’ Motivated Processing of Anti-tobacco Commercials. Health Commun. 2016;32(11):1319–1330. 10.1080/10410236.2016.1220042 27690639

[55] Haley R, Staffaroni J, Fox A. The missing measures of copy testing. J Advert Res. 1994;34(3):46–61.

[56] Harrison D, McLaughlin M. Cognitive processes in self-report responses: Tests of item context effects in work attitude measures. J Appl Psychol. 1993;78(1):129–140. 10.1037/0021-9010.78.1.129 8449851

[57] LeDoux J. Emotion Circuits in the Brain. Annu Rev Neurosci. 2000;23(1):155–184. 10845062 10.1146/annurev.neuro.23.1.155

[58] Chaiken S, Trope Y. Dual-process theories in social psychology. New York: Guilford Press; 1999.

[59] Kaye S, White M, Lewis I. The use of neurocognitive methods in assessing health communication messages: A systematic review. J Health Psychol. 2016;22(12):1534–1551. 10.1177/1359105316630138 26908587

[60] Zelinková J, Shaw D, Mareček R, Mikl M, Urbánek T, Havlíčková D et al An evaluation of traffic-awareness campaign videos: empathy induction is associated with brain function within superior temporal sulcus. Behav Brain Funct. 2014;10(1):27. 25118071 10.1186/1744-9081-10-27 PMC4149038

[61] Falk E, O’Donnell M, Tompson, Gonzalez R, Dal Cin S, Strecher V et al Functional brain imaging predicts public health campaign success.Soc Cogn Affect Neurosci. 2015;11(2):204–214. 10.1093/scan/nsv108 26400858 PMC4733336

[62] Maison D, Oleksy T. Validation of EEG as an Advertising Research Method: Relation Between EEG Reaction Toward Advertising and Attitude Toward Advertised Issue (Related to Political and Ideological Beliefs) In: Nermend K, Łatuszyńska M, ed. by. Neuroeconomic and Behavioral Aspects of Decision Making. Cham: Springer; 2017 p. 273–291.

[63] Piwowarski M. Cognitive neuroscience techniques in examining the effectiveness of social advertisements In: Nermend K, Łatuszyńska M, ed. by. Neuroeconomic and Behavioral Aspects of Decision Making. Cham: Springer; 2017 p. 341–352.

[64] Ordoñana J, González-Javier F, Espín-López L, Gómez-Amor J. Self-Report and Psychophysiological Responses to Fear Appeals. Hum Commun Res. 2009;35(2):195–220.

[65] Boshoff C, Toerien L. Subconscious responses to fear-appeal health warnings: An exploratory study of cigarette packaging. South African Journal of Economic and Management Sciences. 2017;20(1).

[66] Droulers O, Gallopel-Morvan K, Lacoste-Badie S, Lajante M. The influence of threatening visual warnings on tobacco packaging: Measuring the impact of threat level, image size, and type of pack through psychophysiological and self-report methods. PLoS One. 2017;12(9):e0184415 10.1371/journal.pone.0184415 28910317 PMC5598963

[67] Hamelin N, Moujahid O, Thaichon P. Emotion and advertising effectiveness: A novel facial expression analysis approach. Journal of Retailing and Consumer Services. 2017; 36:103–111.

[68] Suckfüll M, Reuter M. Emotions elicited by a road safety campaign. Int J Commun Health. 2013; 2:64–69.

[69] Kessels L, Ruiter R. Eye movement responses to health messages on cigarette packages. BMC Public Health. 2012;12(1). 10.1186/1471-2458-12-352 PMC3464951 22583956

[70] Kessels L, Ruiter R, Wouters L, Jansma B. Neuroscientific evidence for defensive avoidance of fear appeals. Int J Psychol. 2014;49(2):80–88. 10.1002/ijop.12036 24811878 PMC4286019

[71] Wang A, Romer D, Elman I, Turetsky B, Gur R, Langleben D. Emotional graphic cigarette warning labels reduce the electrophysiological brain response to smoking cues. Addict Biol. 2013;20(2):368–376. 10.1111/adb.12117 24330194 PMC4492684

[72] Davidson R, Ekman P, Saron C, Senulis J, Friesen W. Approach-withdrawal and cerebral asymmetry: Emotional expression and brain physiology: I. J Pers Soc Psychol. 1990;58(2):330–341. 2319445

[73] Amodio D, Master S, Yee C, Taylor S. Neurocognitive components of the behavioral inhibition and activation systems: Implications for theories of self-regulation. Psychophysiology. 2007;45(1):11–19. 10.1111/j.1469-8986.2007.00609.x 17910730

[74] Harmon-Jones E, Gable P, Peterson C. The role of asymmetric frontal cortical activity in emotion-related phenomena: A review and update. Biol Psychol. 2010;84(3):451–462. 10.1016/j.biopsycho.2009.08.010 19733618

[75] Ohme R, Reykowska D, Wiener D, Choromanska A. Application of frontal EEG asymmetry to advertising research. J Econ Psychol. 2010;31(5):785–793.

[76] Boksem M, Smidts A. Brain Responses to Movie Trailers Predict Individual Preferences for Movies and Their Population-Wide Commercial Success. J Mark Research. 2015;52(4):482–492.

[77] Vecchiato G, Toppi J, Astolfi L, De Vico Fallani F, Cincotti F, Mattia D et al Spectral EEG frontal asymmetries correlate with the experienced pleasantness of TV commercial advertisements. Med Biol Eng Comput. 2011;49(5):579–583. 10.1007/s11517-011-0747-x 21327841

[78] Cartocci G, Maglione A, Modica E, Rossi D, Cherubino P, Babiloni F. Against smoking public service announcements, a neurometric evaluation of effectiveness. Front Hum Neurosci. 2016;10.

[79] Cartocci G, Modica E, Rossi D, Cherubino P, Maglione A, Colosimo A et al Neurophysiological Measures of the Perception of Antismoking Public Service Announcements Among Young Population. Front Hum Neurosci. 2018;12. 10.3389/fnhum.2018.00231 PMC6124418 30210322

[80] HajiHosseini A, Rodríguez-Fornells A, Marco-Pallarés J. The role of beta-gamma oscillations in unexpected rewards processing. NeuroImage. 2012;60(3):1678–1685. 10.1016/j.neuroimage.2012.01.125 22330314

[81] Kawasaki M, Yamaguchi Y. Frontal theta and beta synchronizations for monetary reward increase visual working memory capacity. Soc Cogn Affect Neurosci. 2012;8(5):523–530. 10.1093/scan/nss027 22349800 PMC3682435

[82] Mas-Herrero E, Ripollés P, HajiHosseini A, Rodríguez-Fornells A, Marco-Pallarés J. Beta oscillations and reward processing: Coupling oscillatory activity and hemodynamic responses. NeuroImage. 2015;119:13–19. 10.1016/j.neuroimage.2015.05.095 26070260

[83] Engel A, Fries P. Beta-band oscillations—signalling the status quo?. Curr Opin Neurobiol. 2010;20(2):156–165. 10.1016/j.conb.2010.02.015 20359884

[84] Marco-Pallarés J, Camara E, Münte T, Rodríguez-Fornells A. Neural Mechanisms Underlying Adaptive Actions after Slips. J Cogn Neurosci. 2008;20(9):1595–1610. 10.1162/jocn.2008.20117 18345985

[85] HajiHosseini A, Holroyd C. Erratum: Reward feedback stimuli elicit high-beta EEG oscillations in human dorsolateral prefrontal cortex. Sci Rep. 2015;5(1). 10.1038/srep13021 PMC4538377 26278335

[86] Cauberghe V, De Pelsmacker P, Janssens W, Dens N. Fear, threat and efficacy in threat appeals: Message involvement as a key mediator to message acceptance. Accid Anal Prev. 2009;41(2):276–285. 10.1016/j.aap.2008.11.006 19245886

[87] De Pelsmacker P, Janssens W. The effect of norms, attitudes and habits on speeding behavior: Scale development and model building and estimation. Accid Anal Prev. 2007;39(1):6–15. 10.1016/j.aap.2006.05.011 16890180

[88] Falk B, Montgomery H. Developing traffic safety interventions from conceptions of risks and accidents. Transp Res Part F Traffic Psychol Behav. 2007;10(5):414–427.

[89] Taubman—Ben-Ari O. The effects of positive emotion priming on self-reported reckless driving. Accid Anal Prev. 2012;45:718–725. 10.1016/j.aap.2011.09.039 22269562

[90] Ruiter R, Kok G, Verplanken B, Brug J. Evoked fear and effects of appeals on attitudes to performing breast self-examination: an information-processing perspective. Health Educ Res. 2001;16(3):307–319. 10.1093/her/16.3.307 11497114

[91] Cho H, Witte K. A review of fear-appeal effects. In: Seiter J, Gass R, ed. by. Perspectives on persuasion, social influence, and compliance gaining. Boston: Allyn and Bacon; 2004 p. 223–238.

[92] Carrera P, Muñoz D, Caballero A. Mixed Emotional Appeals in Emotional and Danger Control Processes. Health Commun. 2010;25(8):726–736. 10.1080/10410236.2010.521914 21153989

[93] Borawska A, Maison D. Impact of negative emotions on public awareness campaigns effectiveness–measuring dilemmas In: Nermend K, Łatuszyńska M, ed. By. Problems, Methods and Tools in Experimental and Behavioral Economics–Computational Methods in Experimental Economics, Springer Proceedings in Business and Economics. Cham: Springer; 2018 p. 113–125,

[94] Ekman P. An argument for basic emotions. Cogn Emot. 1992;6(3–4):169–200.

[95] A fake DUI Car Crash [Internet]. Abingdon: Alamy; c1999-2020 [cited 2020 Mar 15]. Available from: shorturl.at/jrtFP.

[96] Lang P, Bradley M, Cuthbert B. International affective picture system (IAPS): affective ratings of pictures and instruction manual. Gainesville: University of Florida; 2008.

[97] Klem G, Luders H, Jasper H, Elger C. The ten-twenty electrode system of the International Federation. Electroencephalogr Clin Neurophysiol. 1999;52 3:3–6. 10590970

[98] Nitschke J, Miller G, Cook E. Digital filtering in EEG/ERP analysis: Some technical and empirical comparisons. Behav Res Methods Instrum Comput. 1998;30(1):54–67.

[99] Castellanos N, Makarov V. Recovering EEG brain signals: Artifact suppression with wavelet enhanced independent component analysis. J Neurosci Methods. 2006;158(2):300–312. 10.1016/j.jneumeth.2006.05.033 16828877

[100] Murugappan M, Nagarajan R, Yaacob S. Discrete wavelet transform based selection of salient EEG frequency band for assessing human emotions In: Discrete Wavelet Transforms-Biomedical Applications. IntechOpen, 2011.

[101] Tullett A, Harmon-Jones E, Inzlicht M. Right frontal cortical asymmetry predicts empathic reactions: Support for a link between withdrawal motivation and empathy. Psychophysiology. 2012;49(8):1145–1153. 10.1111/j.1469-8986.2012.01395.x 22646720

[102] Reason J, Manstead A, Stradling S, Baxter J, Campbell K. Errors and violations on the roads: a real distinction?. Ergonomics. 1990;33(10–11):1315–1332. 10.1080/00140139008925335 20073122

[103] Aberg L, Rimmo P. Dimensions of aberrant driver behaviour. Ergonomics. 1998;41(1):39–56. 10.1080/001401398187314 9468806

[104] Niezgoda M, Kamiński T, Kruszewski M, Tarnowski A. Self-Reported Drivers’ Behaviour: An Application of DBQ in Poland. Journal of KONES Powertrain and Transport. 2013;20(1):233–238.

[105] Shaver P, Schwartz J, Kirson D, O'Connor C. Emotion knowledge: Further exploration of a prototype approach. J Pers Soc Psychol. 1987;52(6):1061–1086. 10.1037//0022-3514.52.6.1061 3598857

[106] Davidson R. What does the prefrontal cortex “do” in affect: perspectives on frontal EEG asymmetry research. Biol Psychol. 2004;67(1–2):219–234. 10.1016/j.biopsycho.2004.03.008 15130532

[107] Vecchiato G, Maglione A, Cherubino P, Wasikowska B, Wawrzyniak A, Latuszynska A et al Neurophysiological Tools to Investigate Consumer’s Gender Differences during the Observation of TV Commercials. Comput Math Methods Med. 2014; 2014:1–12. 10.1155/2014/912981 PMC4134790 25147579

[108] Chen F, Peng H, Ma X, Liang J, Hao W, Pan X. Examining the safety of trucks under crosswind at bridge-tunnel section: A driving simulator study. Tunn Undergr Sp Tech. 2019;92:103034.

[109] Hayes A. Introduction to Mediation, Moderation, and Conditional Process Analysis, Second Edition New York: Guilford Publications; 2017.

[110] Carey R, McDermott D, Sarma K. The Impact of Threat Appeals on Fear Arousal and Driver Behavior: A Meta-Analysis of Experimental Research 1990–2011. PLoS One. 2013;8(5): e62821 10.1371/journal.pone.0062821 23690955 PMC3656854

[111] Hastings G, Stead M, Webb J. Fear appeals in social marketing: Strategic and ethical reasons for concern. Psychol Mark. 2004;21(11):961–986.

[112] Basil D, Ridgway N, Basil M. Guilt and giving: A process model of empathy and efficacy. Psychol Mark. 2007;25(1):1–23.

[113] Maison D, Gregg A. Capturing the consumer’s unconscious. Applying the implicit association test in consumer research In: Jansson-Boyd C, Zawisza M, ed. by. Routledge International Handbook of Consumer Psychology. London: Routledge. Taylor&Francis Group; 2016 p. 143–164.

[114] Hakim A, Klorfeld S, Sela T, Friedman D, Shabat-Simon M, Levy D. More is Better: Using Machine Learning Techniques and Multiple EEG Metrics to Increase Preference Prediction Above and Beyond Traditional Measurements. bioRxiv. 2018; 317073.

[115] Cunillera T, Fuentemilla L, Periañez J, Marco-Pallarès J, Krämer U, Càmara E et al Brain oscillatory activity associated with task switching and feedback processing. Cogn Affect Behav Neurosci. 2011;12(1):16–33. 10.3758/s13415-011-0075-5 22160843

